# Entomological assessment of tsetse-borne trypanosome risk in the Shimba Hills human-wildlife-livestock interface, Kenya

**DOI:** 10.3389/fvets.2022.931078

**Published:** 2022-08-16

**Authors:** Faith I. Ebhodaghe, Armanda D. S. Bastos, Michael N. Okal, Daniel K. Masiga

**Affiliations:** ^1^International Centre of Insect Physiology and Ecology, Nairobi, Kenya; ^2^Department of Zoology and Entomology, University of Pretoria, Pretoria, South Africa

**Keywords:** epidemiology, trypanosomiasis, *Glossina*, National Reserve, East Africa

## Abstract

Shimba Hills is a wildlife area in Kenya and a major focus of tsetse-borne trypanosomes in East Africa. In Shimba Hills, tsetse-borne trypanosomes constrain animal health and smallholder livelihoods. However, epidemiological data to guide hotspot-targeted control of infections are limited. This study assessed the dynamics of tsetse-borne trypanosome risk in Shimba Hills with the objective to describe infection hotspots for targeted control. Tsetse flies (*n* = 696) collected in field surveys between November 2018 and September 2019 in Shimba Hills were characterized for chronological age and phenotypic sizes and screened for trypanosome and cattle DNA. Entomological inoculation rates for trypanosome risk assessment were derived from the product of fly abundance and molecular rates of vector infection and confirmed cattle bloodmeals in tsetse flies. In addition, cattle health indicators including anemia scores were assessed in contemporaneous parasitological surveys that screened livestock blood samples (*n* = 1,417) for trypanosome using the buffy-coat technique. Compared with *Glossina brevipalpis* and *G. austeni, G. pallidipes* was the most abundant tsetse fly species in Shimba Hills and had a wider spatial distribution and greater likelihood for infectious bites on cattle. The risk of cattle infection was similar along the Shimba Hills human-wildlife-livestock interface and high within one thousand meters of the wildlife reserve boundary. Trypanosomes in tsetse flies were highly diverse and included parasites of wild-suids probably acquired from warthogs in Shimba Hills. Age and phenotypic sizes were similar between tsetse fly populations and did not affect the probability of infection or cattle bloodmeals in the vectors. Anemia was more likely in trypanosome-positive cattle whilst parasitological infection rates in cattle samples maintained a weak relationship with entomological inoculation rates probably because of the limited time scale of sample collection. Trypanosome risk in Shimba Hills is high in locations close to the wildlife reserve and driven by *G. pallidipes* infectious bites on cattle. Therefore, trypanosome vector control programmes in the area should be designed to reduce *G. pallidipes* abundance and tailored to target sites close to the wildlife reserve.

## Introduction

African trypanosomiasis is a neglected tropical disease of humans and animals caused by tsetse-borne trypanosomes in sub-Saharan Africa. The disease is also known as sleeping sickness in humans and *nagana* in livestock. Humans and animals are exposed to African trypanosomiasis when bitten by tsetse flies that are positive for matured trypanosome parasites in the metacyclic stage of development (1). The epidemiological risk of trypanosome infections from tsetse flies is spatially heterogenous in many African trypanosomiasis endemic foci and largely determined by the extent of tsetse-trypanosome interactions and the frequency of tsetse-host contacts (2). However, field studies that investigate tsetse-trypanosome interactions are limited. Moreover, there are yet fewer studies that explore tsetse-host interactions, particularly in wildlife areas such as Shimba Hills (Kenya) where Channumsin et al. (3) observed human and wildlife but not cattle bloodmeals in tsetse flies.

Shimba Hills is a major tsetse-borne trypanosome hotspot in East Africa and one of the areas in the sub-region where trypanosomes constrain agricultural production activities and rural livelihoods (4–7). Epidemiological surveys in Shimba Hills report trypanosome infection rates of ~50.00% in cattle populations (6). However, rates of trypanosome infections in cattle are widely heterogeneous in Shimba Hills (8) therefore implying that cattle in the area are exposed to variable spatial risk of trypanosome infections from tsetse flies.

Understanding trypanosome spatial risk patterns in the Shimba Hills wildlife area will assist in identifying epidemiological hotspots where cattle are exposed to a high risk of infection. However, trypanosome epidemiology and transmission risk patterns are largely understudied in Shimba Hills. Although different studies in the area have assessed trypanosome diversity and rates in tsetse flies (9–11), none of these studies extended the entire stretch of the human-wildlife-livestock interface or evaluated infection rates in tsetse flies in relation to vector abundance and cattle bloodmeals to determine entomological inoculation rates of trypanosome infections.

Cattle trypanosome infection rates and tsetse entomological inoculation rates maintained significantly positive relationships in Zaire, Gabon, Côte d'Ivoire, and Ethiopia (12). In Eastern Zambia, Mweempwa et al. (13) observed that tsetse entomological inoculation rates were influenced by the vector demographics. The investigators found that anthropogenic pressures affected the age structure of tsetse flies across landscapes experiencing varying levels of vegetation fragmentation. Older female individuals dominated populations of tsetse flies in markedly anthropised locations. According to Mweempwa et al. (13), high entomological inoculation rates, hence the high incidence of cattle infections in markedly anthropised study sites where tsetse flies were sparse, were due to the high proportion of older tsetse flies in those areas.

Indeed, studies show that vector intrinsic traits such as age and also sex are important determinants of arthropod-vector competence in pathogen transmission (10, 14). However, conflicting information exists in field studies regarding the effect of sex on trypanosome risk in tsetse flies. For example, Channumsin et al. (10) discovered a higher trypanosome infection rate in male tsetse flies in Sampu in southern Kenya while Isaac et al. (15) found a higher rate of infection in female tsetse flies in Yankari in northern Nigeria. According to Isaac et al. (15), an average longer lifespan of tsetse flies probably increased the risk of infection in female fly individuals in Yankari. However, data describing the relationship between age and infection risk in tsetse flies under natural conditions are limited. It is also widely accepted that phenotypic body sizes of arthropod-vectors influence pathogen transmission (16, 17); however, investigations of the epidemiological importance of this intrinsic trait in tsetse flies are limited, particularly in the context of variable anthropogenic pressures which are reported to alter environmental resources of tsetse flies and thus drive changes in the vector phenotypic sizes (18).

This study characterized tsetse entomological inoculation rates in the Shimba Hills human-wildlife-livestock interface with the aim of identifying trypanosome hotspots for targeted vector control. To better understand factors influencing trypanosome dynamics, we investigated the effect of non-uniform anthropogenic pressures on tsetse flies across landscapes in Shimba Hills and assessed the implications for cattle trypanosome parasite risk in the area. This work reports the most extensive survey on trypanosome infections in tsetse flies in Shimba Hills. It is also the first to evaluate trypanosome entomological inoculation rates for tsetse fly species in Shimba Hills, as well as the first to systematically assess the relationship between trypanosome entomological risk and cattle parasitological infection rates.

## Materials and methods

### Ethical consent

The study received ethical clearance from the Kenyan National Commission for Science, Technology, and Innovation (License No. NACOSTI/P/20/7344) and the Pwani University Ethics Review (approval number ERC/EXT/002/2020). Field collections of tsetse flies were carried out in collaboration with the Kenya Wildlife Service (KWS), the Kenya Tsetse and Trypanosomiasis Eradication Council (KENTTEC), and local communities in Shimba Hills. Verbal consent was sought and obtained from cattle owners before the collection of blood samples from animals. Technical field staff made every effort to minimize pain and discomfort to animals during blood sample collection. Positive cases of trypanosome infections in animals were treated using diminazene diaceturate (Veriben^®^ manufactured in France by Ceva Sante Animale) and without payments from owners.

### Study area

**Figure 1:**
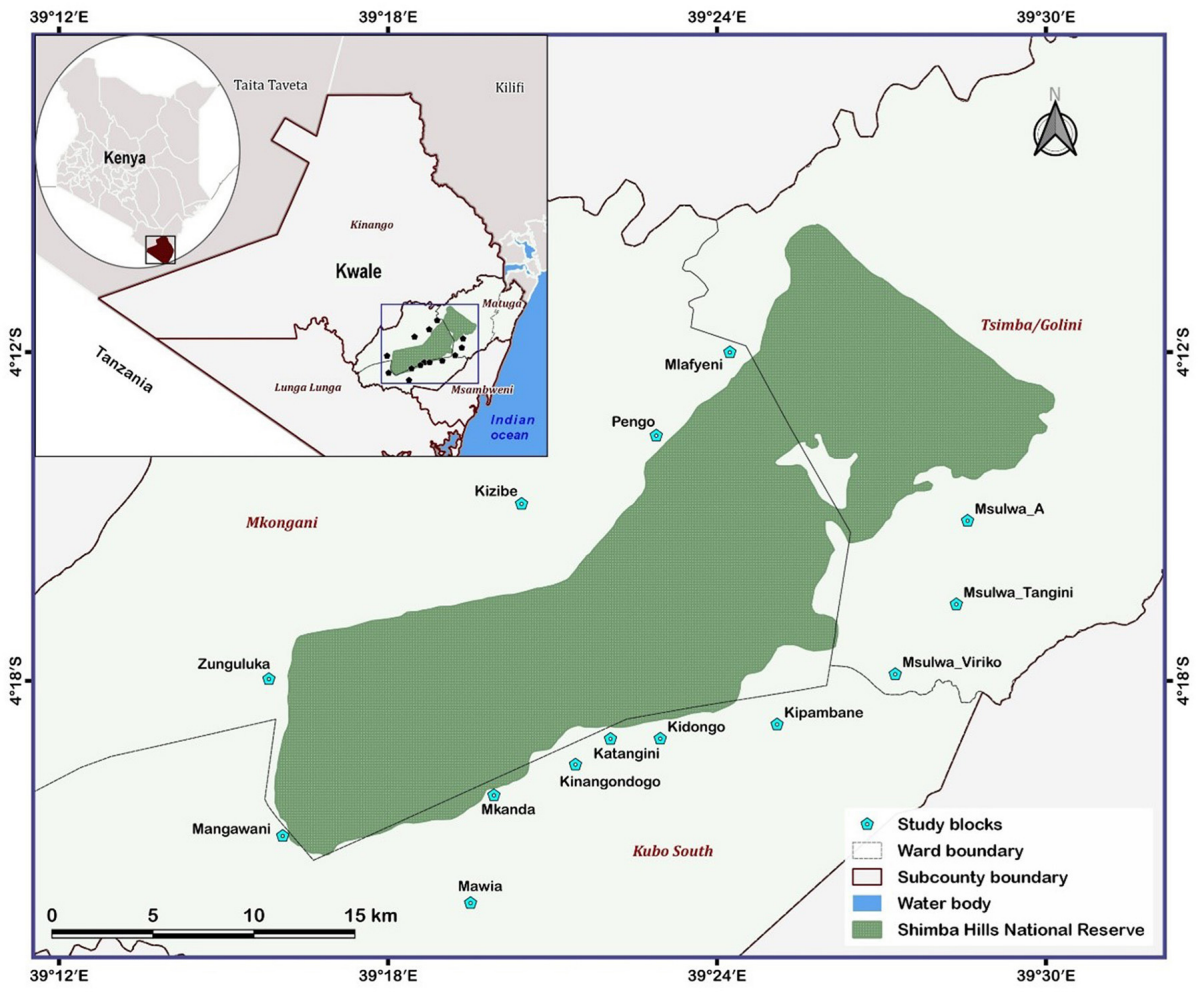
Map showing the 14 study blocks situated within 5 km of the Shimba Hills National Reserve, Kwale county, Kenya.

Shimba Hills where the present study was conducted is a wildlife area located in Kwale County, southeast Kenya (latitude: −4.174°S and longitude: 39.4602 °E) (Figure 1). The area is unique for its high elephant (*Loxodonta africana*) density and extensive faunal diversity including rare and endangered species such as the sable antelope *Hippotragus niger* [(19), Kenya Wildlife Service KWS 2021, http://www.kws.go.ke/content/shimba-hills-national-reserve, accessed December 16, 2021].

The average annual rainfall and temperature in Shimba Hills are 1,150 mm and ~24 °C, respectively. Rainfall in the area is bimodal with long rains from March to May (occasionally extending to July), and short rains from October to December. Vegetation in Shimba Hills ranges from savannah woodlands to shrubby forests and open grasslands interspersed with trees, shrubs, and thickets. The human population in communities surrounding the Shimba Hills National Reserve (SHNR) is ~300,000 people, many of whom are farmers engaging in food crop and livestock production [https://watertowers.go.ke/wp-content/uploads/2020/11/SHIMBA-Hills-Status-Report.pdf, assessed December 16, 2021]. Livestock management in local communities in Shimba Hills is extensive with cattle grazing activities occurring mostly within the area throughout the year.

### Collection and characterization of tsetse flies

Tsetse flies were surveyed in the Shimba Hills human-wildlife-livestock interface using odor-baited biconical traps (20). Biconical traps were baited using cow urine and acetone at respective release rates of 1000 mg/hr and 500 mg/hr. Collections of tsetse flies were carried out over a 231 km^2^ area. The entire area was partitioned into 14 blocks (Figure 1) and each block was further partitioned into grid-cells of 1 km^2^. A biconical trap was deployed within each grid-cell totaling 231 and records were taken of tsetse presence or absence in traps. The spatial distribution of tsetse flies in km square was inferred based on the number of traps that caught at least one tsetse fly throughout the sampling period. Each trap was used to represent one km square. Tsetse flies were collected bimonthly from November 2018 to September 2019 and in different vegetation landscapes and locations within 5 km of the wildlife reserve. However, initial collections (November 2018 to April 2019) were limited to three blocks from Figure 1 (Mlafyeni, Pengo, and Kizibe) but thereafter extended to an additional 11 blocks. During a sampling exercise, collections of tsetse flies were carried out over a four-days period and the vector abundance was expressed as the number of flies per trap per day. Tsetse flies harvested from traps were morphologically identified, sorted according to sex and species using taxonomic keys (21), and preserved in 95% ethanol. Tsetse fly samples for analyses were randomly selected from the total collections in traps. The number of fly samples selected per trap depended on the total collections made. On average, 6 fly individuals were selected per trap. This summed up to 696 tsetse flies caught in 113 biconical traps spread across the entire study period and 14 blocks. Right wings were later carefully detached from each of the 696 tsetse flies. Each wing was assessed for serrations on the trailing edge and the extent of serrations was scored on a scale of 1 to 6 to assess the age of tsetse flies based on the wing fray scoring technique developed by Jackson (22). The number on the scale increased with the age of tsetse flies. Linear measurements were also taken on each wing as a proxy for tsetse fly phenotypic size following the procedure adopted by Hargrove et al. (23).

### Molecular identification of cattle bloodmeals in tsetse flies

Each of the 696 tsetse flies assessed for age and phenotypic sizes were screened for cattle bloodmeals. Cattle DNA extraction was carried out using Genomic DNA extraction kits (Bioloine, London, UK) according to the manufacturer's instructions for animal tissues. Individual tsetse flies were sterilized in alcohol, air-dried, and crushed using a Mini-Beadbeater-16 (BioSpec, Bartlesville, OK, USA). Two vertebrate mitochondrial genes were then amplified in separate Polymerase Chain Reactions (PCRs): i) the 16S ribosomal RNA gene amplified with Vert 16S For: 5′-GAGAAGACCCTRTGGARCTT-3′ and Vert 16S Rev: 5′-CGCTGTTATCCCTAGGGTA-3′ primers targeting an ~200 bp region (24), and ii) the cytochrome *b* gene amplified with the Cyt b For: 5′-CCCCTCAGAATGATATTTGTCCTCA-3′ and Cyt b Rev: 5′-CATCCAACATCTCAGCATGATGAAA-3′ primers targeting an ~383 bp region (25). Each PCR-reaction contained 0.5 μM of each Forward and Reverse primer (Macrogen, Europe, Amsterdam, The Netherlands), 1 μL template DNA, and 2 μL of pre-formulated 5X HOT FIREPol^®^ EvaGreen^®^ HRM Mix, (Solis BioDyne, Tartu, Estonia) in a 10 μL reaction-volume. DNA amplifications were carried out for 16S ribosomal RNA and cytochrome *b* respectively in a Rotor-Gene Q thermocycler (Qiagen, Hilden, Germany) and QuantStudio 3 Real-Time PCR System thermal cycler (MicroAmp^®^ Applied Biosystems, Inc., Foster City, CA, USA). Thermal cycling conditions for DNA amplification were: initial denaturation for 15 min at 95 °C, followed by 40 cycles of denaturation at 95 °C for 40 s, annealing at 56 °C for 20 s, and extension at 72 °C for 30 s, and a final extension at 72 °C for 5 min. High-Resolution Melting analysis of amplicons followed immediately with gradual melting from 75 to 95 °C. A non-template (negative) control was included in each PCR-HRM run. Cattle DNA in tsetse flies was identified by comparing melting profiles for alignment with HRM profiles of cattle DNA positive controls. Melting profiles were analyzed in the software Rotor-Gene Q v2.1 and QuantStudioTM Design & Analysis v1.5.1 depending on the machine used for PCR-HRM analysis. Amplification and amplicon sequencing of the CO1 gene (26) were carried out to confirm positive cases of cattle bloodmeals in tsetse flies. An ~750 bp region of the CO1 gene was targeted for amplification with 0.5 μM of each Forward and Reverse primer (Macrogen, Europe) (VF1d For: TCTCAACCAACCACAARGAYATYGG; VR1d Rev: TAGACTTCTGGGTGGCCRAARAAYCA) (26) in a 15 μL reaction-volume containing, 2 μL template DNA, 3 μL of 5X HOT FIREPol^®^ Blend Master Mix (Solis BioDyne, Tartu, Estonia). Cycling conditions for the amplification were: initial denaturation for 15 min at 95 °C, followed by 40 cycles of denaturation at 95 °C for 20 s, annealing at 57 °C for 30 s, and extension at 72 °C for 60 s, followed by a final extension at 72 °C for 7 min. The success of DNA amplification was ascertained by electrophoresis of PCR products for 30 min in a 1.5% agarose gel stained with 5 μg/mL ethidium bromide at 120 V. Unincorporated dNTPs and PCR primers were removed from amplicons using Exo-SAP (USB Corporation, Cleveland, OH, USA). Purified amplicons were then submitted for unidirectional Sanger sequencing at Macrogen in Europe.

### Molecular detection and characterization of trypanosomes in tsetse flies

The same DNA extracts prepared from tsetse fly homogenates using the Genomic DNA extraction kits (Bioloine, London, UK) for cattle bloodmeal analysis were screened for trypanosome DNA. A segment of the Internal Transcribed Spacer (ITS) region of the trypanosome genome was amplified using 0.5 μM of each of Forward and Reverse ITS-1 primers (CF: CCGGAAGTTCACCGATATTG, BR: TTGCTGCGTTCTTCAACGAA) (27) in a 10 μL reaction-volume containing 1 μL DNA template, and 5 μL DreamTaq Master Mix (2X) (Thermo Scientific, UK). Cycling conditions for trypanosome ITS-1 DNA amplification were: initial denaturation for 1 min at 95 °C, followed by 35 cycles of denaturation at 95 °C for 30 s, annealing at 60 °C for 20 s, and extension at 72 °C, and a final extension at 72 °C for 7 min. Amplicons were sized against a molecular weight maker (Gene-Ruler 100 bp DNA ladder, Thermo Scientific, Lithuania) on a 1.5% agarose gel stained with ethidium bromide (5 μg/mL). The following unique band sizes were used to characterize trypanosomes: *T. vivax* ~250 bp, *T. godfreyi* ~300 bp, *T. simiae* Tsavo ~370 bp, *T. simiae* ~400 bp, Trypanozoon (*T. brucei* sp.) ~480 bp, *T. congolense* Kilifi ~620 bp, and *T. congolense* Savannah/Forest ~700 bp (27). Further analyses to confirm trypanosome identity were carried out based on amplicon sequencing. Cleaning of amplicon to remove unincorporated dNTPs and PCR primers was performed using Exo-SAP (USB Corporation, Cleveland OH) and purified products were submitted for unidirectional Sanger sequencing at Macrogen in Europe.

### Trypanosome parasitological surveys in cattle

Cattle in Shimba Hills were screened for trypanosomes at two different seasons during entomological surveys. The first screening was carried out in June 2019 while the second screening was carried out in September-October 2019. Cattle recruitment was by the single-stage household-cluster sampling technique. For the parasitological survey, cattle were assembled in central crush-pens in each of the 14 blocks where tsetse flies were collected. Cattle were pricked on their ear veins using sterilized lancets and blood samples were collected into capillary tubes for trypanosome examination in the buffy coat (28). Most blood sample collections in Shimba Hills were done in the morning but sometimes extended into the afternoons. Cattle were also assessed for anemia based on the Packed Cell Volume (PCV) using a microhaematocrit reader (Hawksley Ltd., UK). Body weight was estimated based on heart girth measurements using calibrated bands (6).

### Data analyses

Cattle and trypanosome DNA sequence chromatograms were inspected for quality, edited in the BioEdit software v7.2.5 (29), and submitted to BLAST analysis for comparison to nucleotide sequences in the NCBI GeneBank-*nr* database (https://blast.ncbi.nlm.nih.gov/Blast.cgi). Sequences were identified based on a homology cut-off of 99.00 to 100% for cattle DNA sequences and 80.00 to 100% for trypanosome DNA sequences. Trypanosome DNA sequence alignments were implemented online in Clustal Omega (https://www.ebi.ac.uk/Tools/msa/clustao/) and the unaligned regions were trimmed off before further analyses in the *MEGA-X* software (30). A Maximum-Likelihood phylogenetic tree to show trypanosome diversity was estimated based on 1000 bootstrap replications using default parametres in *MEGA-X*. The Smart Model Selection in PhyLM (31) selected the Hasegawa-Kishino-Yano HKY model of sequence evolution (32) as the best-fit model used in tree construction. The tree was rooted using a sequence of the Kinetoplastid *Bodo caudatus* (GenBank accession number: AY028450).

Statistical analyses were conducted in the *R* statistical environment (33). Negative Binomial Generalized Linear Mixed Models (NB-GLMMs) (34) with ‘*trap_ID*' as random-effect were used to assess significant differences in the abundance of tsetse flies with ‘sex', ‘species', and ‘collection site' (landscape vegetation and distance from wildlife reserve) as predictor variables. Entomological risk (Entomological Inoculation Rate EIR) of cattle trypanosome infections was expressed as the product of tsetse abundance and rates of infection and confirmed cattle bloodmeals in sampled tsetse flies. EIRs were multiplied by 365 to derive annual [*a*]-EIRs. Mean *a-*EIRs were used to estimate the average number of trypanosome-positive tsetse flies expected to feed on cattle per year in Shimba Hills.

Mean *a*-EIRs, Wing Fray Scores (WFS), and phenotypic sizes of tsetse flies were significantly different from a normal distribution (*P* < 0.05) using the Shapiro-Wilk's test. Consequently, a-EIRs, WFS, and phenotypic size variations between tsetse flies were assessed using the non-parametric Mann-Whitney *U* test to examine for significant differences between fly sex and the Kruskal-Wallis test to examine for significant differences between fly species, landscape vegetation and distance from wildlife reserve. Probabilities of tsetse infection and cattle bloodmeals in tsetse flies were assessed in Binomial-GLMMs using ‘*trap_ID*' as random-effect and ‘fly_sex', ‘fly_species', ‘fly_WFS' and ‘fly_phenotypic_size' as predictor variables.

Differences between cattle sex, trypanosome species, and blocks in the proportion of cattle infection (infected vs. uninfected) were assessed for significance using Binomial-Generalized Linear Models (B-GLMs) (34). Cattle PCVs and girth measurements were significantly different from a normal distribution (*P* < 0.05) using the Shapiro-Wilk's test. Therefore, comparisons of mean PCVs and mean girth measurements between infected and uninfected cattle were done using the Mann-Whitney *U* test. The Spearman Correlation Coefficients (*rho*) were calculated to assess the relationship between mean *a*-EIRs and cattle trypanosome infection rates across blocks. An *alpha* level of 0.95 was selected in all analyses. Where significant differences were detected following a Kruskal-Wallis test, pairwise comparisons were carried out based on the Dunn's *post-hoc* test (35). Furthermore, Tukey's *post-hoc* tests were carried out in the '*multcomp'* R package (36) for GLMMs and GLMs having two or more predictor variables and *P* < 0.05.

## Results

We collected a total of 10,996 tsetse flies in the entomological survey in Shimba Hills. This comprised of 22.45% males (*n* = 2,469) and 77.55% females (*n* = 8,527). Morphological identification confirmed that *G. pallidipes* was the most abundant species (95.11%, *n* = 10,458), followed by *G. brevipalpis* (3.58%, *n* = 394) and *G. austeni* (1.31%, *n* = 144). Almost all tsetse flies (96.71%, 10,634/10,996) collected in the entomological survey at the human-wildlife-livestock interface were trapped in sites within 1000 m from the SHNR. *Glossina austeni* were trapped only within 1000 m of the Shimba Hill NR. For the other species, tsetse fly abundance decreased with distance from the reserve, irrespective of sex. Male and female tsetse flies were respectively collected in 44% (102 km^2^) and 62% (143 km^2^) of the entire 231km^2^ area surveyed while *G. pallidipes* were collected in 61% (140 km^2^), *G. austeni* in 15% (35 km^2^) and *G. brevipalpis* in 25% (58 km^2^) of the same area.

### Abundance of tsetse flies across landscapes

**Figure 2:**
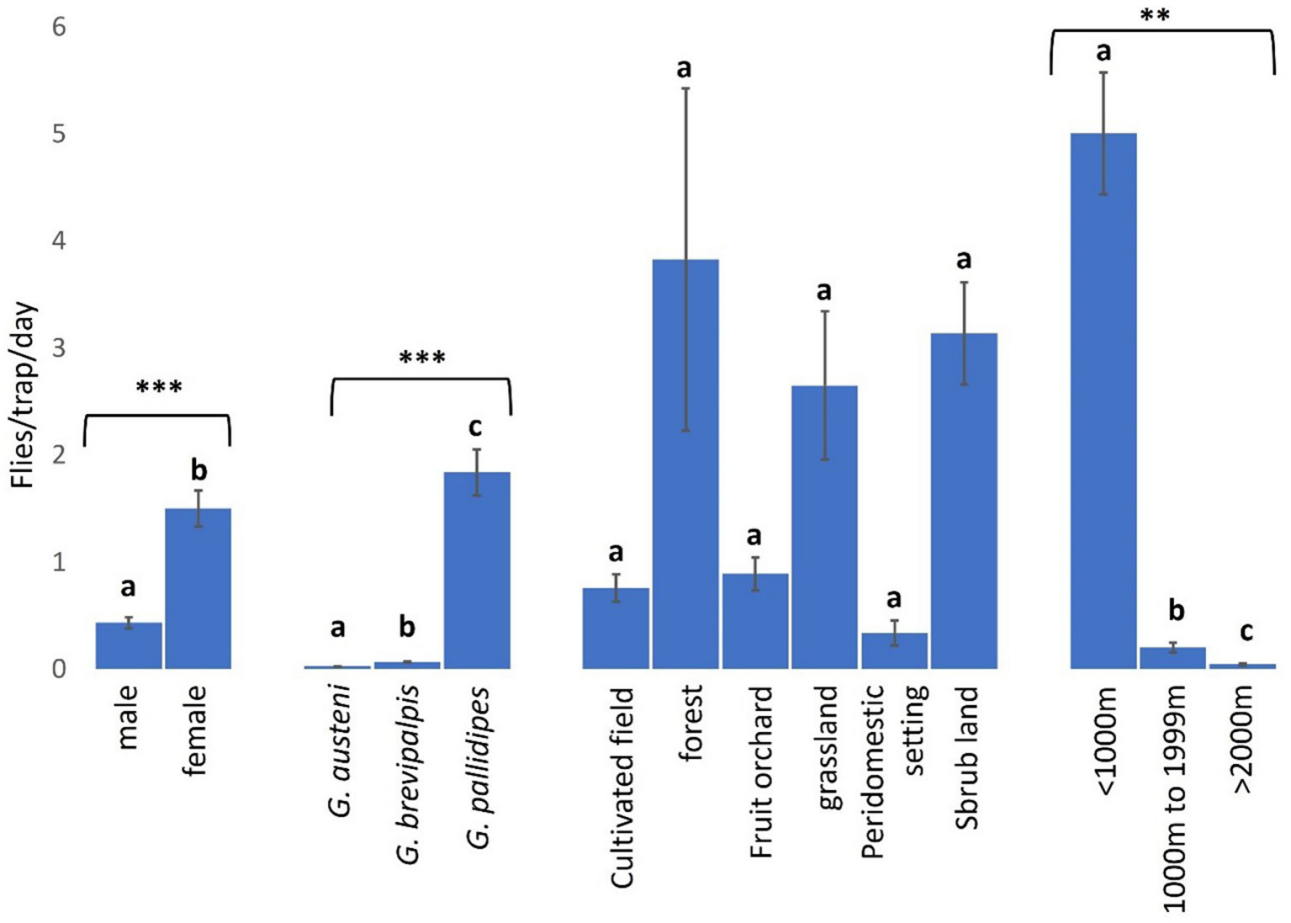
Abundance of tsetse flies according to fly sex and species, and collection sites (landscape type and proximity to Shimba Hills National Reserve). *** and ** correspond to *P* values < 0.0001 and <0.01 respectively. Error bars are used to indicate the standard error of the mean (SEM). Letters have been used to indicate presence or absence of significant difference in pairwise comparisons of tsetse fly abundance. Pairwise comparisons are made between tsetse flies within the same group defined by sex, species, vegetation site or distance from the wildlife reserve. Significantly different pairs are denoted using different letters while insignificantly different pairs are indicated using same letters.

Female tsetse flies (1.50 FTD. 95% CI: 1.17 to 1.83) were significantly more abundant than males (0.43 FTD. 95% CI: 0.33 to 0.54) (NB-GLMM: *P* < 0.0001), and *G. pallidipes* (1.84 FTD. 95% CI: 1.42 to 2.26) was significantly more abundant than *G. brevipalpis* (0.07 FTD. 95% CI: 0.06 to 0.08) and *G. austeni* (0.03 FTD. 95% CI: 0.02 to 0.03) (NB-GLMM: *P* < 0.0001) (Figure 2).

Forested areas (3.83 FTD. 95% CI: 0.66–4.49) had the highest abundance of tsetse flies among vegetation landscapes, but this was not significantly different from the vector abundance in the other locations (NB-GLMM: *P* > 0.05), including cultivated fields (0.76 FTD. 95% CI: 0.51–1.27) and peridomestic settings (0.34 FTD. 95% CI: 0.10–0.44) where tsetse fly abundance was least (Figure 2). Tsetse abundance was significantly higher within 1000 m (5.00 FTD. 95% CI: 3.89–8.89) of the reserve than in other areas 1000–1999 m (0.20 FTD. 95% CI: 0.11–0.32) and >2000 m (0.04 FTD. 95% CI: 0.03–0.07) from the reserve (NB-GLMM: *P* < 0.01).

### Epidemiological importance of tsetse flies

**Figure 3:**
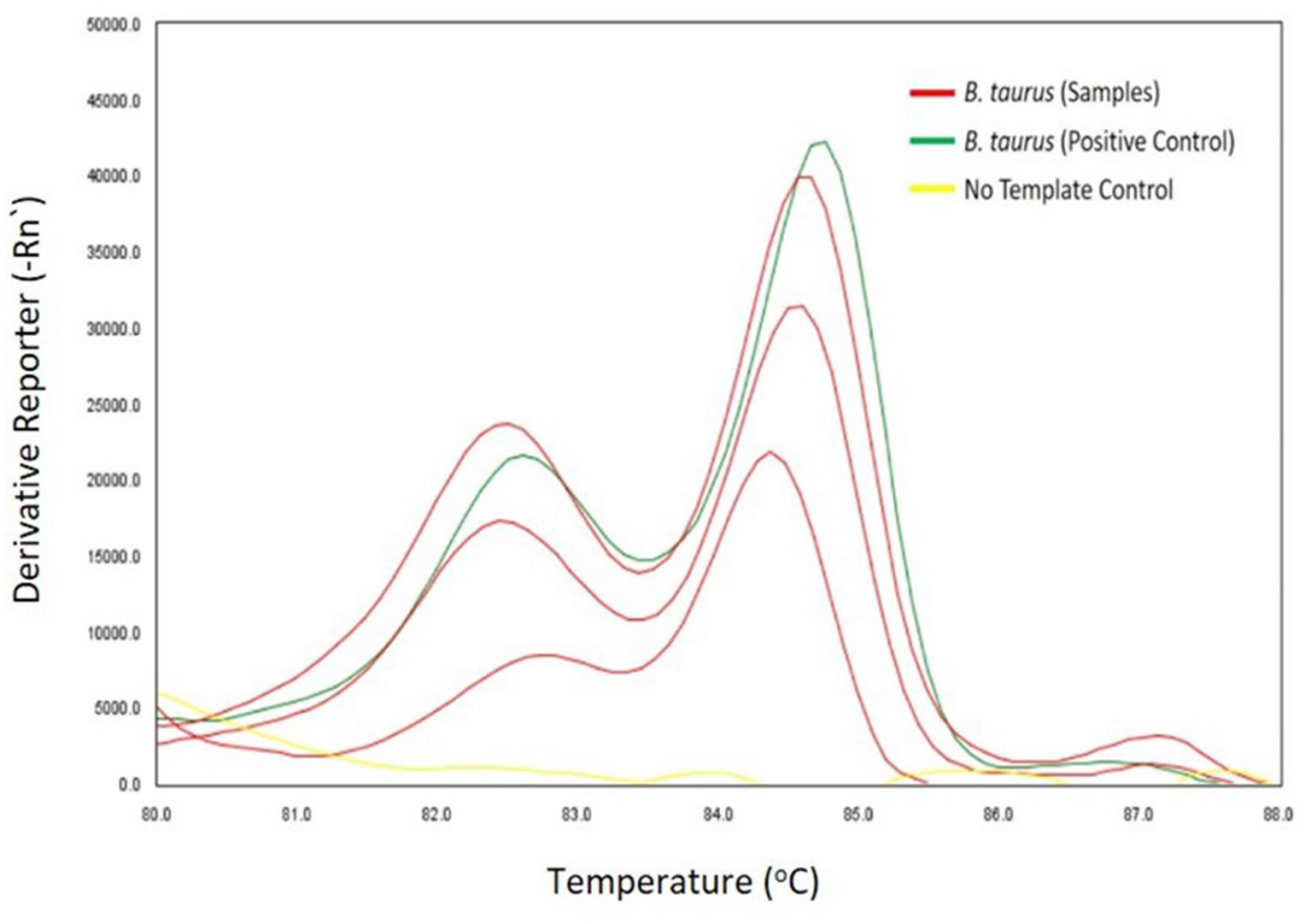
HRM profiles showing melting curves of cattle DNA.

**Figure 4:**
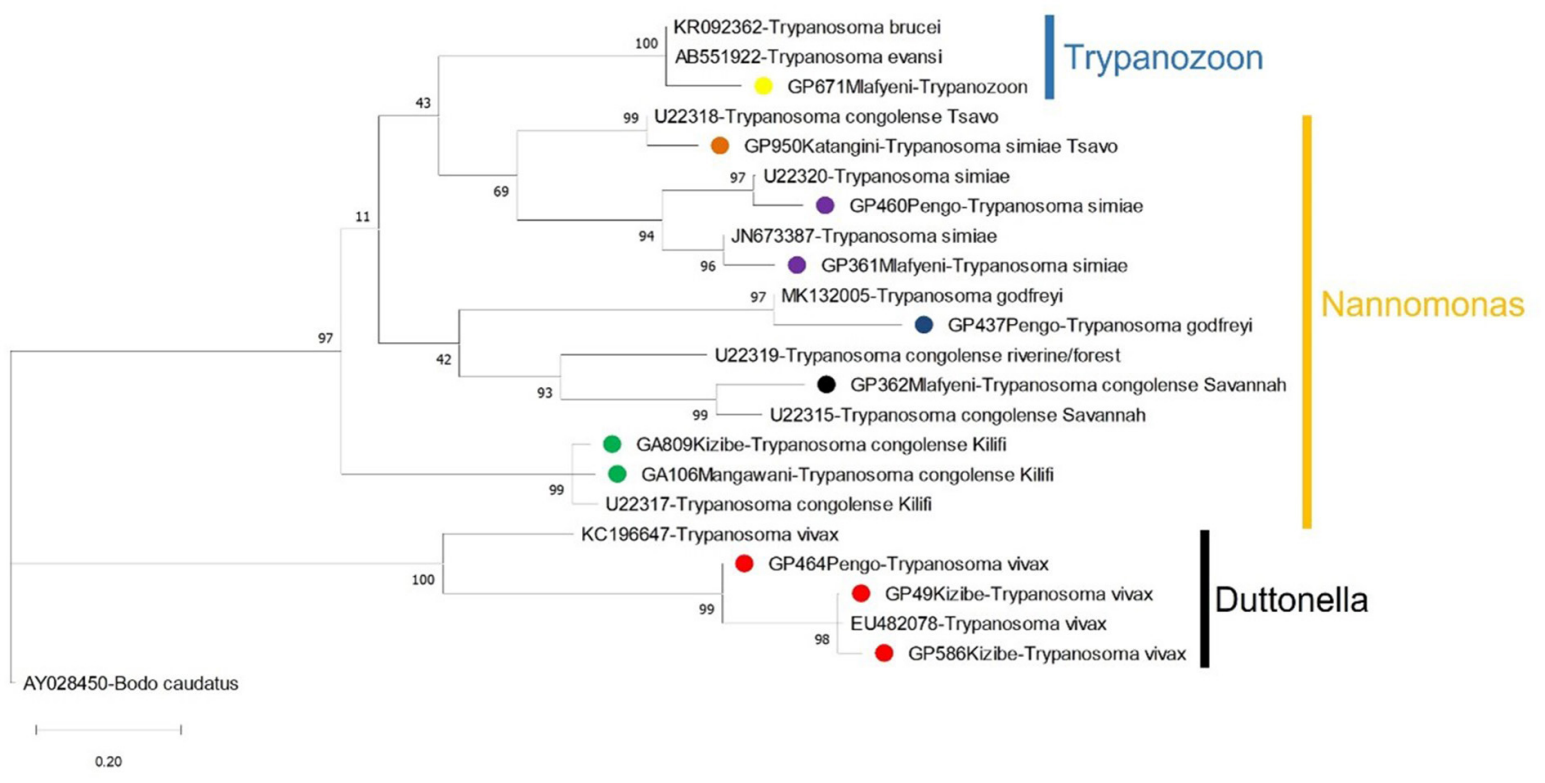
A Maximum-Likelihood phylogenetic tree showing the species of trypanosomes detected in tsetse flies in Shimba Hills in Kenya (November 2018 to September 2019). Sequences from the study are bulleted using different colors to indicate trypanosome parasites. Other sequences shown on the tree were obtained from GenBank. Vertical bars are used to depict the subgenera of trypanosomes. Nodal support values based on 1000 bootstrap replicates are indicated next to each node. The branch length represents substitution per site.

Out of 696 tsetse flies screened in molecular analyses, 11.35% (95% CI: 8.99–13.71) and 8.62% (95% CI: 6.53–10.71) were respectively positive for cattle bloodmeals (Figure 3) and trypanosome infections (Figure 4, Table 1), GenBank Accession Numbers: OM942761 — OM942765, MW689623—MW689625, OM937961, OM914942, OM914939). Among the trypanosome species identified in tsetse flies, *T. vivax* (2.44%. 95% CI: 1.29–3.59) was the most prevalent (Table 1). Furthermore, tsetse flies were positive for the double infections *T. congolense* Savannah and *T. brucei sl*. (0.29%. 95% CI: −0.11–0.69), *T. congolense* Kilifi and *T. congolense* Savannah (0.14%. 95% CI: −0.14–0.43) and *T. brucei sl*. and *T. vivax* (0.14%. 95% CI: −0.14–0.43) and the triple infections *T. simiae, T. simiae* Tsavo and *T. godfreyi* (0.14%. 95% CI: −0.14–0.43) and *T. simiae, T. simiae* Tsavo and *T. vivax* (0.14%. 95% CI: −0.14–0.43).

**Table 1:** Identification of trypanosome nucleic acid sequences recovered from tsetse fly samples in Shimba Hills (2018 to 2019).

**Sample ID**	**Block**	**Latitude**	**longitude**	**Fly species**	**Sex**	**Sequence length (bp)**	**Closest match on GenBank (host, location)**	**Species**	**Sequence identity (%)**
**GP437**	**Pengo**	−4.25076	39.36938	*G. pallidipes*	Female	116	MK131956 (Tsetse fly, Zambia)	*T. godfreyi*	91.67
**GP362**	**Mlafyeni**	−4.2508491	39.36904	*G. pallidipes*	Female	313	U22315 (Rat, Kenya)	*T. congolense* Savannah	94.49
**GA106**	**Mangawani**	−4.35776	39.25443	*G. austeni*	Female	536	MK756200 (Tsetse fly, Nigeria)	*T. congolense* Kilifi	82.51
**GA693**	**Kizibe**	−4.2894689	39.27341	*G. austeni*	Female	459	MK756200 (Tsetse fly, Nigeria)	*T. congolense* Kilifi	82.37
**GA809**	**Kizibe**	−4.2894689	39.27341	*G. austeni*	Female	527	MK756200 (Tsetse fly, Nigeria)	*T. congolense* Kilifi	89.85
**GP671**	**Mlafyeni**	−4.2000908	39.40392	*G. pallidipes*	Male	326	KR092362 (Colobus, Cote d'Ivoire)	*T. brucei sl*	97.45
**GP950**	**Katangini**	−4.3202	39.36612	*G. pallidipes*	Female	340	U22318 (Tsetse fly, Kenya)	*T. simiae* Tsavo	92.46
**GP361**	**Mlafyeni**	−4.1745264	39.39222	*G. pallidipes*	Female	345	JN673387 (Warthog, Tanzania)	*T. simiae*	93.08
**GP460**	**Pengo**	−4.21669	39.3731	*G. pallidipes*	Male	362	U22320 (?, Kenya)	*T. simiae*	91.69
**GP464**	Pengo	−4.24723	39.36326	*G. pallidipes*	Male	221	KX584844 (Tsetse fly, Mozambique)	*T. vivax*	100
**GP49**	Kizibe	−4.27402	39.30951	*G. pallidipes*	Female	209	KX584844 (Tsetse fly, Mozambique)	*T. vivax*	99.42
**GP586**	Kizibe	−4.2715603	39.33925	*G. pallidipes*	Male	208	KX584844 (Tsetse fly, Mozambique)	*T. vivax*	100

**Figure 5:**
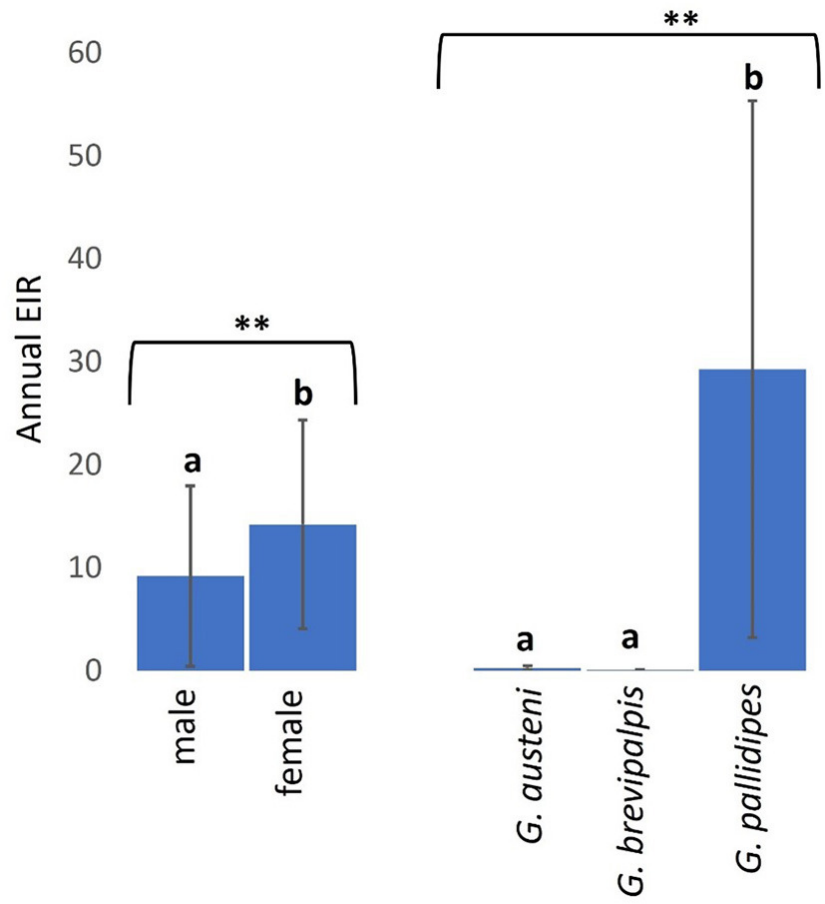
Annual EIRs of tsetse flies according to fly sex and species. Error bars are used to indicate the standard error of the mean (SEM). Letters have been used to indicate presence or absence of significant difference in pairwise comparisons of tsetse fly entomological inoculation rates. Pairwise comparisons are made between tsetse flies within the same group defined by sex or species. Significantly different pairs are denoted using different letters while insignificantly different pairs are indicated using same letters. ** indicates *P* value < 0.01.

Overall, 0.86% (95% CI: 0.17–1.55) of screened tsetse flies were positive for both trypanosome infections and cattle bloodmeals. The overall rate of confirmed cattle bloodmeals was higher in trypanosome-positive female flies (mean *a-*EIR: 14.19. 95% CI: −7.72–36.09) than male flies (mean *a-*EIR: 9.17. 95% CI: −9.78–28.12) (Mann-Whitney *U* test: *P* = 0.0331) and in *G. pallidipes* (mean *a-*EIR: 29.26. 95% CI: −27.10–85.62) than *G. austeni* (mean *a-*EIR: 0.27. 95% CI: −0.19–0.73) and *G. brevipalpis* (mean a-EIR: 0.05. 95% CI:−0.01–0.11) (Figure 5 Kruskal-Wallis test: H = 11.92, *d.f* = 2, *P* < 0.01).

### Spatial entomological risk of cattle trypanosome infections

**Figure 6:**
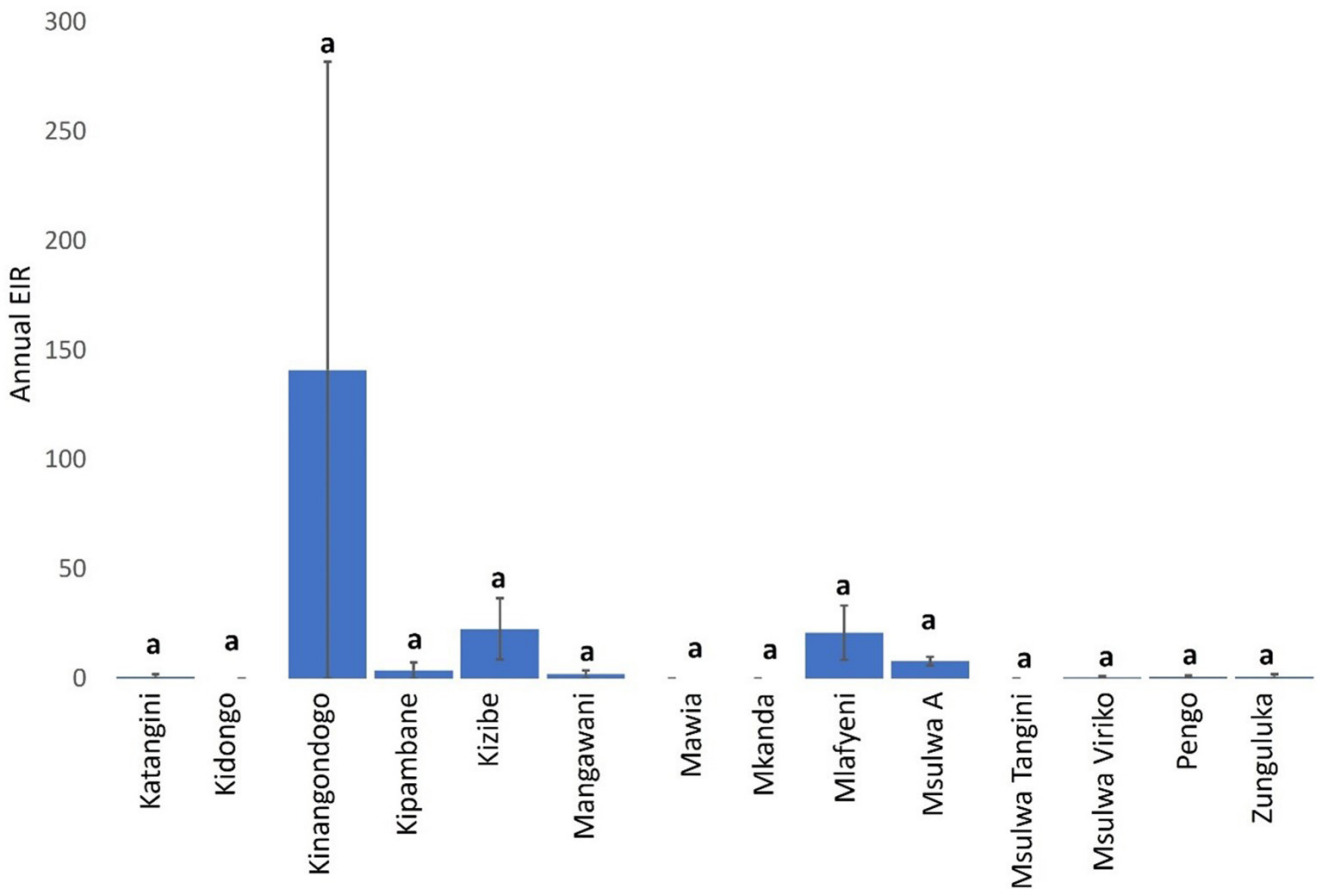
Annual EIR of tsetse flies according to study-block. Error bars are used to indicate the standard error of the mean (SEM). Same letters on top of vertical bars have been used to indicate absence of significant difference in pairwise comparisons of tsetse fly entomological inoculation rates in study blocks.

Mean a-EIR in Shimba Hills was 14.42 (95% CI: −1.65–30.49). Entomological risk of cattle trypanosome infections though relatively high in Kinangodongo (mean a-EIR: 140.89. 95%CI: −1,649.29–1,931.07) was not significantly different between study blocks (Figure 6, Kruskal-Wallis test: H = 14.52, *d.f* = 13, *P* = 0.3385).

**Figure 7:**
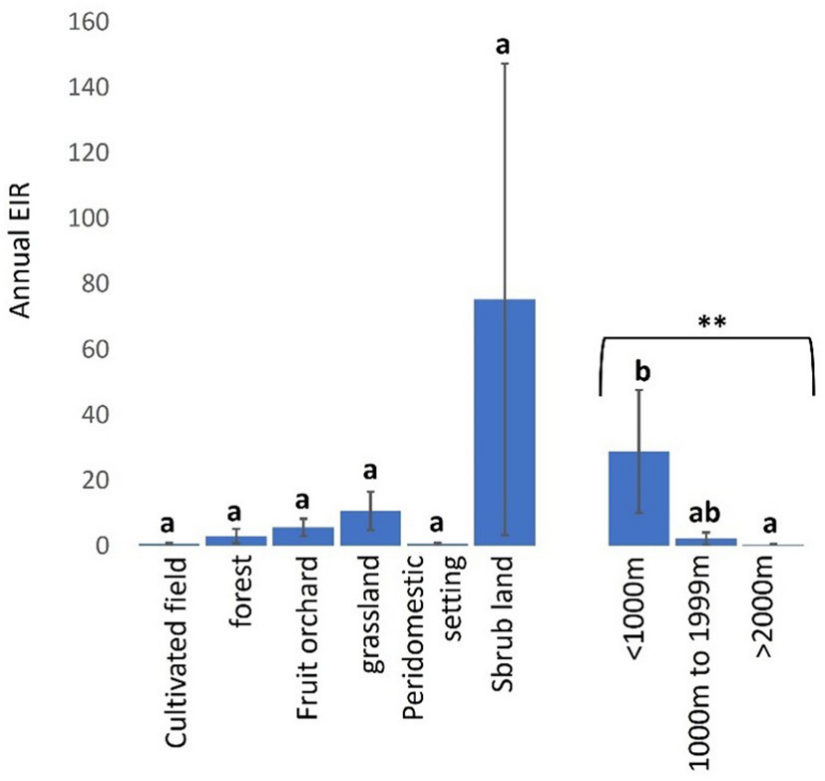
Annual EIR of tsetse flies according to vegetation landscape and proximity to wildlife reserve in Shimba Hills. Error bars are used to indicate the standard error of the mean (SEM). Letters have been used to indicate presence or absence of significant difference in pairwise comparisons of tsetse fly entomological inoculation rates. Pairwise comparisons are made between tsetse flies within the same group defined by vegetation site or distance from the wildlife reserve. Significantly different pairs are denoted using different letters while insignificantly different pairs are indicated using same letters. **Indicates *P* value < 0.01.

Trypanosome-infected tsetse flies fed on cattle more frequently in shrub-lands (mean a-EIR: 75.31. 95% CI: −83.13–233.74) than other landscapes and sparsely in cultivated fields (mean a-EIR: 0.62. 95% CI: −0.10–1.33) and peridomestic settings (mean a-EIR: 0.51. 95% CI: −0.66–1.68) (Figure 7, Kruskal-Wallis test: H = 6.01, *d.f* = 5, *P* = 0.3057). Finally, infected tsetse flies were more likely to feed on cattle in locations within 1000 m (mean a-EIR: 28.75. 95% CI: −11.79–69.28) of the wildlife reserve in Shimba Hills than 1000–1999 m (mean a-EIR: 2.14. 95% CI: −2.16–6.45) and >2000 m (mean a-EIR: 0.30. 95% CI: −0.37–0.97) from the reserve (Figure 7, Kruskal-Wallis test: H = 10.30, *d.f* = 2, *P* < 0.01).

### Age structure of tsetse flies across landscapes

**Figure 8:**
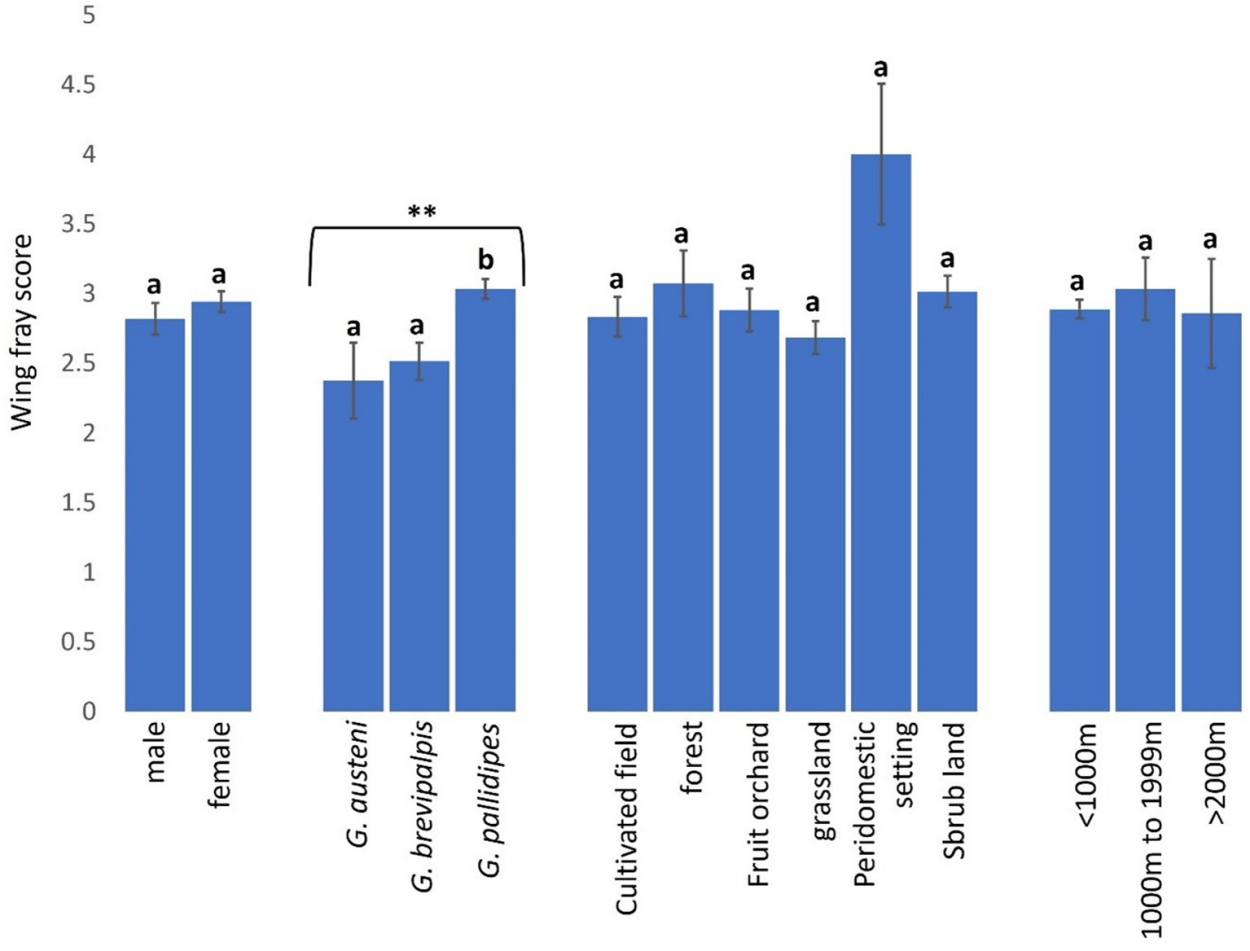
Wing fray scores of tsetse flies according to fly sex and species and collection site. Error bars are used to indicate the standard error of the mean (SEM). Letters have been used to indicate presence or absence of significant difference in pairwise comparisons of tsetse fly wing fray scores. Pairwise comparisons are made between tsetse flies within the same group defined by sex, species, vegetation site or distance from the wildlife reserve. Significantly different pairs are denoted using different letters while insignificantly different pairs are indicated using same letters.

Wing fray scores (WFS) used to assess age of tsetse flies were not significantly higher in female (WFS: 2.94. 95% CI: 2.80–3.09) than male (WFS: 2.82. 95% CI: 2.59–3.04) tsetse flies (Mann-Whitney *U* test: P = 0.09502) but were significantly higher in *G. pallidipes* (WFS: 3.03. 95% CI: 2.89–3.18) than *G. brevipalpis* (WFS: 2.52. 95% CI: 2.25–2.78) and *G. austeni* (WFS: 2.38. 95% CI: 1.82–2.93) (Figure 8, Kruskal-Wallis test: H = 17.63, *d.f* = 2, *P* < 0.01). Tsetse flies were, on average, youngest in grasslands (WFS: 2.68 (95% CI: 2.45–2.92) and oldest in peridomestic settings (WFS: 4.00. 95% CI: 2.88–5.12) than in other landscapes (Figure 8, Kruskal-Wallis test: H = 9.08, *d.f* = 5, *P* = 0.1059). The age of tsetse flies was similar between locations <1000 m [WFS: 2.89 (95% CI: 2.76–3.02), 1000–1999m (WFS: 3.03. 95% CI: 2.58–3.48) and >2000m (WFS: 2.86. 95% CI: 2.04–3.68] from the reserve (Figure 8, Kruskal-Wallis test: H = 0.36, *d.f* =2, *P* = 0.8338).

### Phenotypic sizes of tsetse flies across landscapes

**Figure 9:**
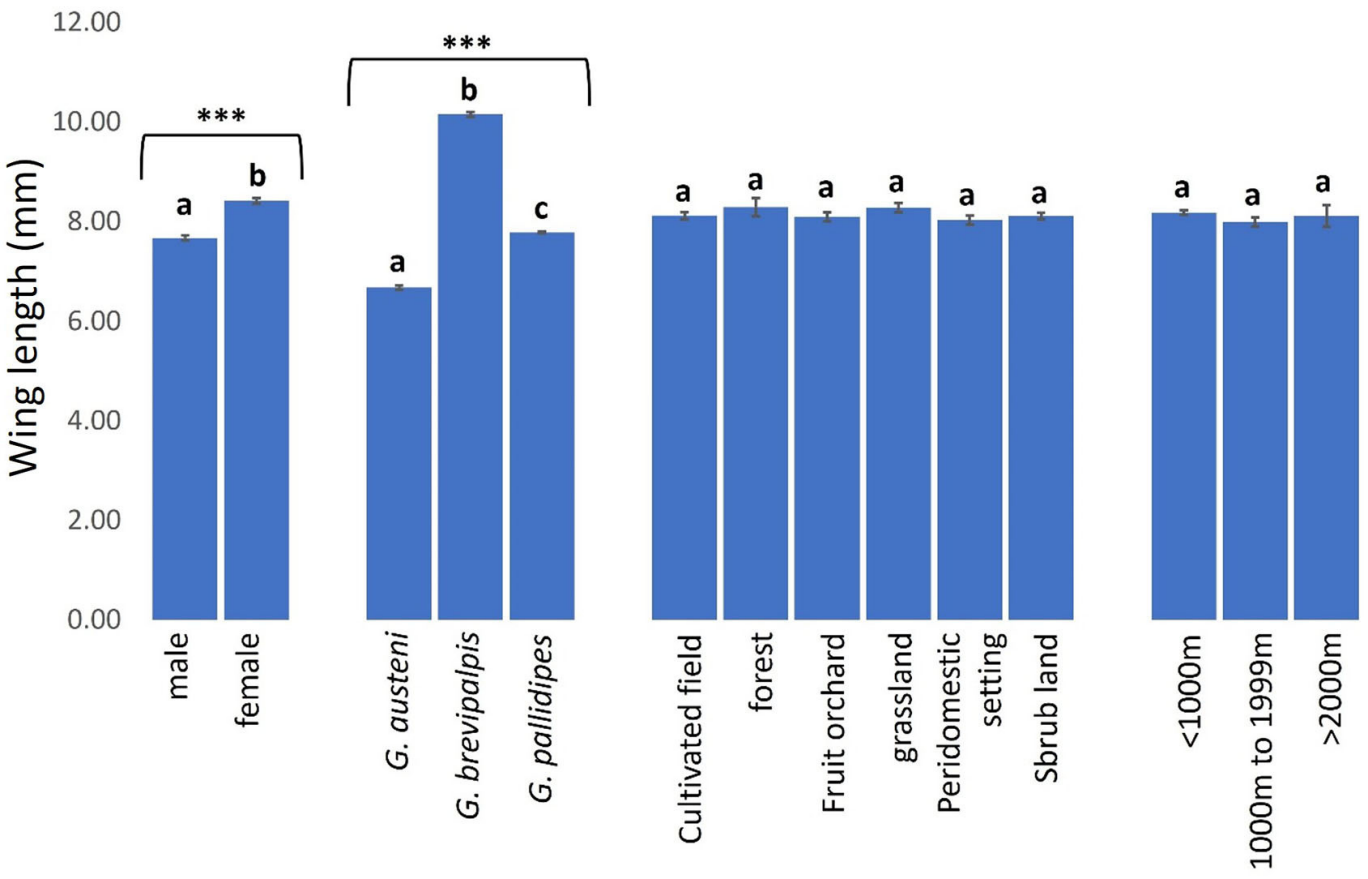
Wing length of tsetse flies according to fly sex and species and collection site. Error bars are used to indicate the standard error of the mean (SEM). Letters have been used to indicate presence or absence of significant difference in pairwise comparisons of tsetse fly wing lengths of tsetse flies. Pairwise comparisons are made between tsetse flies within the same group defined by sex, species, vegetation site or distance from the wildlife reserve. Significantly different pairs are denoted using different letters while insignificantly different pairs are indicated using same letters. **Indicates *P* value < 0.0001.

Tsetse phenotypic sizes were significantly higher in female (8.41 mm. 95% CI: 8.31–8.51) than male (7.67 mm. 95% CI: 7.57–7.77) (Mann-Whitney *U* test: *P* < 0.0001) and in *G. brevipalpis* (10.15 mm. 95% CI: 10.05–10.24) than *G. pallidipes* (7.78 mm. 95% CI: 7.75–7.81) and *G. austeni* (6.68 mm. 95% CI: 6.58–6.77) (Figure 9, Kruskal-Wallis test: H = 288.43, *d.f* = 2, *P* < 0.0001). Phenotypic sizes of tsetse flies were similar between landscapes, ranging from 8.02 mm (95% CI: 7.82–8.23) in peri-domestic settings to 8.29 mm (95% CI: 7.93–8.65) in forests (Figure 9, Kruskal-Wallis test: H = 2.68, *d.f* = 5, *P* = 0.7487). Tsetse phenotypic sizes were also similar between the vector populations in areas <1000 m (8.18 mm. 95% CI: 8.09–8.26), 1000–1999 m (7.99 mm. 95% CI: 7.80–8.18) and >2000 m (8.11 mm. 95% CI: 7.64–8.57) from the reserve (Figure 9, Kruskal-Wallis test: H = 0.03, *d.f* =2, *P* = 0.9842).

### Effects of vector intrinsic traits on trypanosome infections and cattle bloodmeals in tsetse flies

Trypanosome rate was higher in female (0.09. 95% CI: 0.07–0.16) than male (0.07. 95% CI: 0.04–0.11) tsetse flies and in *G. austeni* (0.20. 95% CI: 0.07–0.27) than *G. pallidipes* (0.09. 95% CI: 0.06–0.15) and *G. brevipalpis* (0.05. 95% CI: 0.01–0.06). Tsetse fly species (NB-GLMM: *P* < 0.05) but not sex (Binomial-GLMM: *P* > 0.05) affected the likelihood of the vector infection. Tsetse age (B-GLMM: *P* > 0.05) and phenotypic size (after controlling for fly species effect) (B-GLMM: *P* > 0.05) were also not significantly associated with the probability of trypanosome infection. Furthermore, female tsetse flies (0.13. 95% CI: 0.10–0.24) had a higher rate of cattle bloodmeals than male tsetse flies (0.08. 95% CI: 0.04–0.12). Cattle feeding rates were higher in *G. austeni* (0.18. 95% CI: 0.05–0.23) than *G. pallidipes* (0.11. 95% CI: 0.08–0.19) and *G. brevipalpis* (0.11. 95% CI: 0.05–0.16). However, fly sex (NB-GLMM: *P* < 0.05) but not fly species (B-GLMM: *P* > 0.05) was significantly associated with probability of detecting a cattle bloodmeal in tsetse flies. Neither tsetse age (B-GLMM: *P* > 0.05) nor phenotypic size (B-GLMM: *P* > 0.05) was associated with the probability of cattle bloodmeals in tsetse flies.

### Cattle trypanosome infections and association with trypanosome entomological inoculation rates

**Figure 10:**
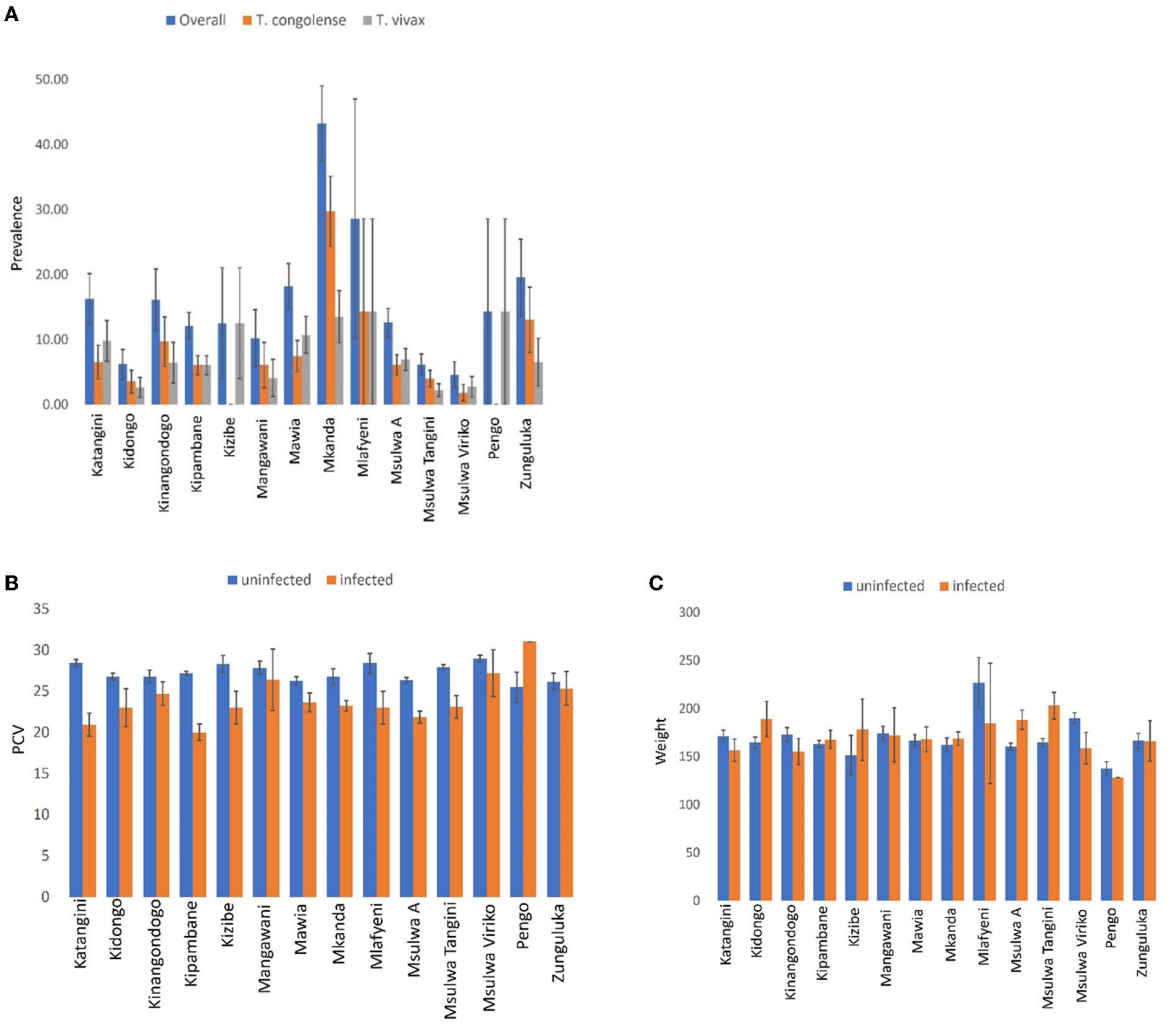
Bar-charts showing: **(A)** Trypanosome infection prevalence, **(B)** packed cell volume (PCV), and **(C)** girth measurements in cattle populations in Shimba Hills. Error bars are used to indicate the standard error of the mean (SEM).

**Figure 11:**
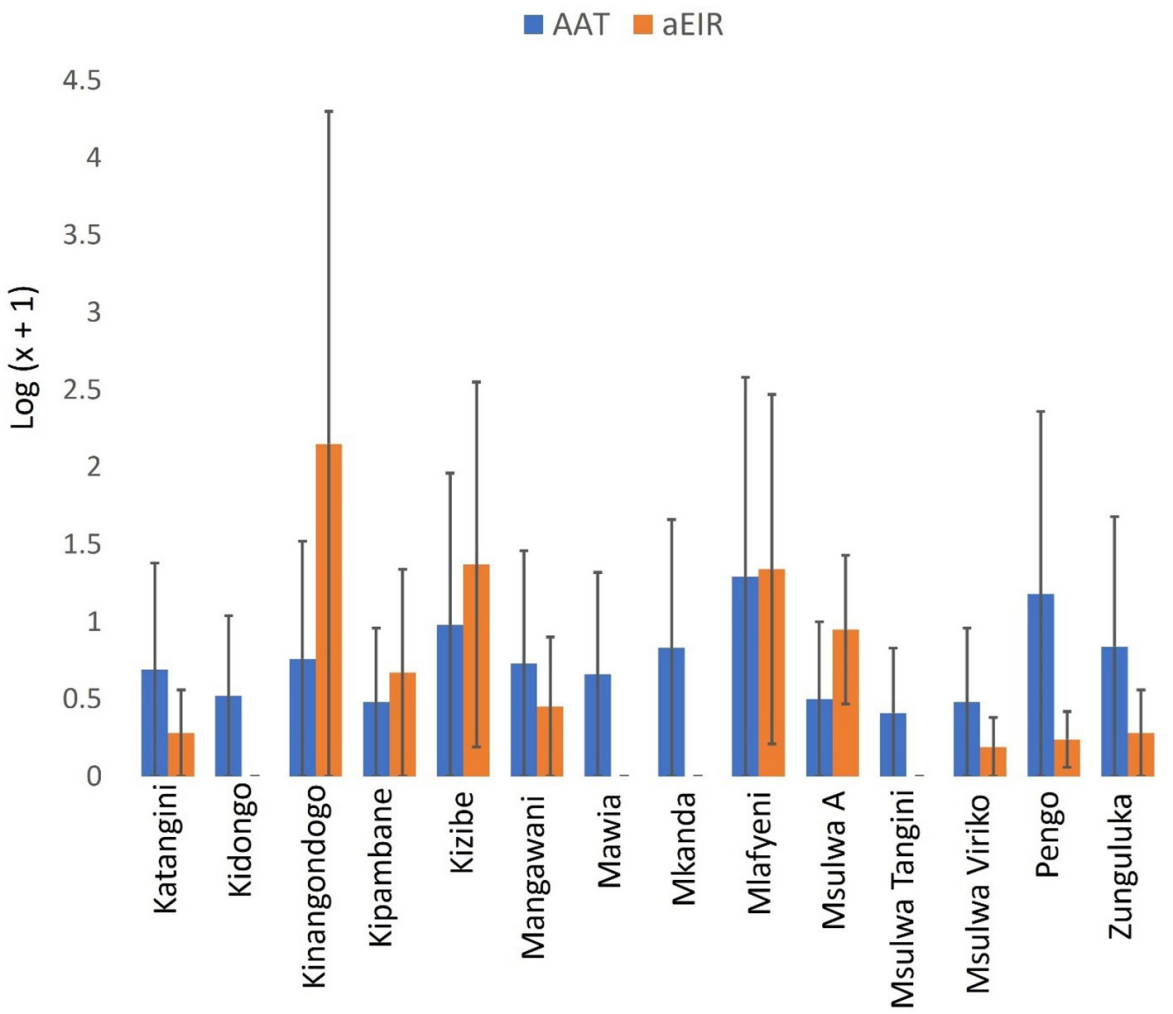
Annual EIR of tsetse flies and trypanosome parasitological rates in cattle. Error bars are used to indicate the standard error of the mean (SEM).

A total of 185 (13.06%. 95% CI: 11.30–14.81) out of 1,417 cattle screened for trypanosomes were positive for infection in Shimba Hills (Figure 10). Male cattle (16.86%. 95% CI: 13.87–19.85) had a significantly higher proportion of infection than female cattle (10.22%. 95% CI: 8.13–12.31) (BGLM: *P* = 0.0003). Cattle were infected with *T. congolense* (6.92%. 95% CI: 5.59–8.24) and *T. vivax* (6.21%. 95% CI: 4.95–7.47) (BGLM: *P* = 0.4483). The proportion of infection was highest in Mkanda (43.24%. 95% CI: 31.69–54.80) and significantly different between study blocks (BGLM: *P* < 0.05). Average Packed Cell Volume was significantly lower in infected (22.71. 95% CI: 21.95–23.47) than uninfected (27.26. 95% CI: 27.00–27.52) cattle (Mann-Whitney *U* test: *P* < 0.0001). However, average girth measurements were similar between infected (173.02 cm. 95% CI: 165.55–180.48) and uninfected (167.03 cm. 95% CI: 163.86–170.19) cattle (Mann-Whitney *U* test: *P* > 0.05). Trypanosome entomological risk across study blocks lacked association with cattle trypanosome infection rates whether overall (Figure 11). Spearman's Correlation Coefficient (*rho* = 0.13 *P* = 0.6657) or during the long rains in May-June (*rho* = −0.02. *P* = 0.9505) or dry season in August-October (*rho* = −0.25. *P* = 0.3817).

## Discussion

The present study provides insights into trypanosome spatial risk in Shimba Hills and reassessed the species diversity and abundance of tsetse flies at the human-wildlife-livestock interface. For the first time, the study provides empirical data to show evidence of ongoing interactions between tsetse flies, trypanosomes, and cattle in Shimba Hills and identified grasslands close to the wildlife reserve as hotspots of cattle infections. The study incriminated female tsetse flies and *G. pallidipes* as being responsible for most cattle trypanosome infections in Shimba Hills and thus corroborates previous reports of female and *G. pallidipes* tsetse flies as epidemiologically important vectors of trypanosomes (37, 38). The epidemiological importance of female tsetse flies and *G. pallidipes* in Shimba Hills is further supported by data from the present work showing extensive distribution and high abundance of these vectors in the area and also showing *G. pallidipes* relatively high average lifespan and female tsetse high likelihood to feed on cattle.

The average annual entomological inoculation rate obtained for tsetse flies in Shimba Hills was 14.42 and indicates that cattle in the area are exposed to bites from one trypanosome-positive tsetse fly every 26 days, an almost two-fold higher rate than the 50 days reported in the Ghibe Valley in Ethiopia (39). However, this frequency of tsetse-cattle contacts in Shimba Hills is an under-estimation considering that tsetse flies in certain locations were characterized to have annual entomological inoculation rates of >14.42 suggesting increased frequent encounters between tsetse flies and cattle in these sites. Kinangodongo, one of the study blocks in Shimba Hills where we assessed tsetse flies for the degree of trypanosome risk posed to cattle, is located close to the Shimba Hills National Reserve precisely within one thousand meters of the wildlife reserve. It was therefore not surprising that Kinangodongo is among study blocks recorded to have the highest average annual entomological inoculation rate of 140.89 implying cattle exposure to attack from one infected tsetse fly at least every 3 days.

*Glossina pallidipes, G. austeni* and *G. brevipalpis* collections in traps show further evidence of the endemicity of these fly species in Shimba Hills (8, 10). Tsetse high infestations close to the wildlife reserve were unequivocally influenced by the vector high abundance within the National Reserve and contributed to the high entomological inoculation rates of cattle trypanosome infections uncovered in sites near the reserve. Studies in the Serengeti National Park in Tanzania (40) and the Akagera National Reserve in Rwanda (41) among other wildlife areas in East Africa (42, 43) reported a high abundance of tsetse flies within wildlife protected areas. However, as with Serengeti and Akagera, the numbers of tsetse flies in Shimba Hills were observed to progressively decline from wildlife protectorates following a decline in vegetation cover providing resting sites as well as animal species providing bloodmeals for the vectors.

To show the effect of vegetation cover and the absence thereof on tsetse flies in Shimba Hills were the findings of a high abundance of tsetse flies in forests and shrub lands and a sparse abundance of the vectors in cultivated fields and peridomestic settings. However, tsetse flies in different taxa respond non-uniformly to disturbances inflicted on the environment by human activities (44–46). *Glossina austeni* tsetse flies, for example, are highly sensitive to environmental disturbances. Therefore, they are reported in only pristine habitats (46). The high sensitivity of *G. austeni* to habitat degradation very likely accounts for the fly species low abundance in Shimba Hills and limited distribution to only areas close to the wildlife reserve where anthropogenic activities are extremely sparse or absent.

Contrary to expectation, populations of tsetse flies across disparately anthropised landscapes had similar wing fray scores and wing lengths. This perhaps is because the surveyed anthropised sites where stress conditions are expected to select for older and phenotypically larger tsetse flies were not sufficiently distant from the wildlife reserve. A study in eastern Zambia that reported a significant difference in the average age of *G. morsitans morsitans* populations collected tsetse flies along a transect of over 20 km stretching from anthropogenically undisturbed Lusandwa to markedly anthropised Chisulo (13). A different study in north-eastern Zambia observed significant variations in phenotypic sizes of tsetse flies collected along a transect line of 15 km in Rufunsa, 45 km in Mpika, and 46 km in Lundazi extending from human residential areas to wildlife protected areas (18). Tsetse flies in Shimba Hills were collected over an area of 5 km from human settlements to the edge of the wildlife reserve. Tsetse flies are probably migrating and mixing freely within this short distance where samplings were done and might explain the homogeneity in age and phenotypic sizes of the vectors observed in Shimba Hills.

Our study successfully characterized a wide diversity of trypanosomes in tsetse flies and is the first single epidemiological study to report up to seven species and subspecies of the parasite in Shimba Hills. The extensive spatio-temporal range of tsetse collection in Shimba Hills and the application of sensitive molecular diagnostic tools for infection detection and characterization allowed us to capture a broad animal trypanosome diversity at the wildlife-livestock interface. The broad diversity of trypanosomes in Shimba Hills portends complex epidemiology for the *nagana* cattle disease caused by tsetse-borne trypanosomes in the area. *Trypanosoma congolense* has been described as a major trypanosome parasite of cattle in Shimba Hills (5, 8). In this study, we were able to characterize two (Savannah and Kilifi) strains of *T. congolense*; the Forest strain of *T. congolense* is primarily endemic to *Palpalis* tsetse infested riverine ecologies in West and Central Africa (15), hence the parasite was not detected in Shimba Hills where *Savannah* tsetse are the main trypanosome vectors; moreover, *T. congolense* Forest is largely absent in East Africa except for sporadic detections made in tsetse flies (47). Additionally, we observed differential clustering with strong bootstrap support for isolates of *T. simiae* (OM942763 and OM937961) and *T. vivax* (MW689624 — MW689625) on the phylogenetic tree. This is indicative of the likely existence of multiple genotypes for each of the trypanosomes in Shimba Hills. Multiple genotypes for *T. simiae* and *T. vivax* could further complicate *nagana* epidemiology in Shimba Hills and thus should be investigated.

Trypanosomes detected in tsetse flies were more diverse close to the wildlife reserve. Among parasites detected in tsetse flies collected close to the reserve were *T. simiae Tsavo* and *T. godfreyi* reported commonly in wildlife and sparsely in livestock. Tsetse flies possibly had acquired these parasites from wildlife bloodmeals in Shimba Hills. A previous work reported wildlife bloodmeals in tsetse flies in Shimba Hills (3). However, samplings were done in Buffalo Ridge within the reserve and Zunguluka along the wildlife interface thus it was not possible to have a clear assessment of animal bloodmeal sources of tsetse flies across the wildlife interface or reliably decipher wildlife sources of trypanosomes in the vectors.

The cattle trypanosome infection rate of 13.06% in Shimba Hills and detection of only two *Trypanosoma* species is likely an underestimation considering that we utilized the buffy coat diagnostic technique unlike a previous epidemiological survey in the area that used sensitive molecular tools to screen cattle bloodmeals for trypanosomes with a report of an infection rate of 32.70% and five *Trypanosoma* species (5). After the PCR-High-Resolution Melting technique was used (Kenya) to screen cattle blood samples, in the Ruma wildlife-livestock interface (Kenya), Kalayou et al. (48) recorded an infection rate of 27.90% with detection of four *Trypanosoma* species, as against 11.00% when the investigators applied the buffy coat diagnostic technique. The application of sensitive tools in subsequent studies in Shimba Hills will not only allow for an accurate and reliable assessment of trypanosome infection rates and species diversity in cattle but could assist with unraveling parasite intraspecific diversity. However, a parallel study is assessing *T. vivax* diversity in the tsetse fly samples analyzed in this study with an objective to provide insights into trypanosome genotype diversity and associated clinical conditions in cattle in the area.

The finding of significantly lower anemia scores in trypanosome-infected than uninfected cattle shows clearly that the parasites are a burden on livestock health in Shimba Hills. However, it was not possible to show a clear relationship between trypanosome parasitological rates of cattle infections and tsetse entomological inoculation rates. Similar studies revealed that spatio-temporal scale of data collection could affect apparent relationships between cattle parasitological rates and trypanosome entomological rates (49, 50). In the Fall et al. (49) study in Senegal, data were collected over a four-year period and a significant association between cattle parasitological rates and trypanosome entomological rates was observed only after aggregating monthly datasets collected over the entire study period and lagging entomological data by 3 months. Bett et al. (50) obtained a statistically significant relationship for both variables in Nkuruman in southwest Kenya after pooling monthly datasets collected over 17 months. In Shimba Hills, logistical challenges constrained parasitological and entomological data collection on a monthly basis and over a longer period; entomological data were collected bimonthly for 10 months and parasitological data were collected once in the long rain season and once in the dry season. Otherwise, it may have been possible to detect significant relationships between cattle parasitological rates and trypanosome entomological rates in Shimba Hills.

## Conclusion

Cattle in Shimba Hills are exposed to a high risk of trypanosome infection from female tsetse flies and *G. pallidipes* in grazing fields close to the wildlife reserve. The present study provides no evidence that landscape anthropisation has an influence on trypanosome risk in the area but shows that tsetse flies exist at high infestation levels close to the wildlife reserve unequivocally on account of favorable living conditions and with the likelihood that the vectors are feeding on wild fauna species in these locations and thus potentially exposing cattle to infections from wildlife reservoirs of trypanosomes. We recommend tsetse control programs in the Shimba Hills wildlife-livestock interface to target operations to trypanosome hotspots close to the National Reserve. Meanwhile, findings from the present study highlight the need for further investigations that screen wildlife for trypanosomes or tsetse flies for animal bloodmeal sources in cattle farming communities in Shimba Hills. This could further improve understanding of trypanosome epidemiology in the area. Further studies spanning several years will be important to better understand the relationship between cattle parasitological rates and trypanosome entomological rates in Shimba Hills.

## Data availability statement

The dataset used and/or analyzed in the present study are available from the corresponding author FIE on reasonable request. DNA sequences of vertebrate species generated during the study are available in the GenBank under accession numbers: OM942761—OM942765, MW689623—MW689625, OM937961, OM914942, and OM914939.

## Ethics statement

The study was reviewed and approved by the Kenyan National Commission for Science, Technology, and Innovation (License No. NACOSTI/P/20/7344) and the Pwani University Ethics Review (Approval No. ERC/EXT/002/2020).

## Author contributions

FIE, MNO, ADSB, and DKM: conceptualization. FIE: data curation, formal analysis, investigation, methodology, and writing–original draft. MNO and DKM: funding acquisition and project administration. ADSB, MNO, and DKM: supervision. FIE, ADSB, and DKM: writing–review and editing. All authors read and approved the manuscript.

## Funding

FIE was supported by a German Academic Exchange Service (DAAD) in-region postgraduate scholarship and registered for his Ph.D. degree at the University of Pretoria, South Africa. We gratefully acknowledge the financial support for this research by the following organizations and agencies: the BioVision Foundation Switzerland (Grant No. DPA_005/2018-31.03.2020); European Union's Integrated Biological Control Applied Research Programme–tsetse repellent component (EU-IBCARP tsetse) awarded to *icipe* (Grant No. DCI-FOOD/2014/346-739); This study also received financial support from the German Ministry for Economic Cooperation and Development (BMZ) through the Deutsche Gesellschaft für Internationale Zusammenarbeit (GIZ) ICTDL Grant No. 18.7860.2-001.00; UK's Department for International Development (DFID); Swedish International Development Cooperation Agency (SIDA); the Swiss Agency for Development and Cooperation (SDC); and the Kenyan Government. The funders had no role in study design, data collection and analysis, decision to publish, or preparation of the manuscript.

## Conflict of interest

The authors declare that the research was conducted in the absence of any commercial or financial relationships that could be construed as a potential conflict of interest.

## Publisher's note

All claims expressed in this article are solely those of the authors and do not necessarily represent those of their affiliated organizations, or those of the publisher, the editors and the reviewers. Any product that may be evaluated in this article, or claim that may be made by its manufacturer, is not guaranteed or endorsed by the publisher.

## Author disclaimer

The views expressed herein do not necessarily reflect the official opinion of the donors.

## Abbreviations

SHNR: Shimba Hills National Reserve
KWS: Kenya Wildlife Service
KENTTEC: Kenya Tsetse and Trypanosomiasis Eradication Council
DNA: Deoxyribo-Nucleic Acid
PCR: Polymerase Chain Reaction
HRM: High Resolution Melting
*icipe*: International Center of Insect Physiology and Ecology
NB-GLMM: Negative Binomial Generalized Linear Mixed Model
B-GLMM: Binomial Generalized Linear Mixed Model
B-GLM: Binomial Generalized Linear Model
PCV: Packed Cell Volume
WFS: Wing Fray Scores
mg/her: Milligram per hour
EIR: Entomological inoculation rate
CI: Confidence interval
NACOSTI: Kenyan National Commission for Science, Technology and Innovation.
